# Dynamic distribution of gut microbiota in cattle at different breeds and health states

**DOI:** 10.3389/fmicb.2023.1113730

**Published:** 2023-02-16

**Authors:** Lei Wang, Daoyi Wu, Yu Zhang, Kun Li, Mingjin Wang, Jinping Ma

**Affiliations:** ^1^Bijie Institute of Animal Husbandry and Veterinary Science, Bijie, China; ^2^College of Veterinary Medicine, Huazhong Agricultural University, Wuhan, China; ^3^College of Veterinary Medicine, Institute of Traditional Chinese Veterinary Medicine, Nanjing Agricultural University, Nanjing, China

**Keywords:** Weining cattle, Angus cattle, diarrhea, gut microbiota, 16S

## Abstract

Weining cattle is a precious species with high tolerance to cold, disease, and stress, and accounts for a large proportion of agricultural economic output in Guizhou, China. However, there are gaps in information about the intestinal flora of Weining cattle. In this study, high-throughput sequencing were employed to analyze the intestinal flora of Weining cattle (WN), Angus cattle (An), and diarrheal Angus cattle (DA), and explore the potential bacteria associated with diarrhea. We collected 18 fecal samples from Weining, Guizhou, including Weining cattle, Healthy Angus, and Diarrheal Angus. The results of intestinal microbiota analysis showed there were no significant differences in intestinal flora diversity and richness among groups (*p* > 0.05). The abundance of beneficial bacteria (*Lachnospiraceae*, *Rikenellaceae*, *Coprostanoligenes*, and *Cyanobacteria*) in Weining cattle were significantly higher than in Angus cattle (*p* < 0.05). The potential pathogens including *Anaerosporobacter* and *Campylobacteria* were enriched in the DA group. Furthermore, the abundance of *Lachnospiraceae* was very high in the WN group (*p* < 0.05), which might explain why Weining cattle are less prone to diarrhea. This is the first report on the intestinal flora of Weining cattle, furthering understanding of the relationship between intestinal flora and health.

## Introduction

Weining is located in southwest China at an average altitude of 2,200 meters. It is a large livestock county where cattle raising is the main economic income of farmers. In 2021, the stock of cattle in Weining was 153,600, accounting for 6.5% of the local agricultural output, with the most farmed species being Weining cattle and Angus cattle. The Weining cattle are an ancient ruminant with hypoxia tolerance, anti-oxidant action, and disease resistance, and have become the symbol of Weining Guizhou. They resemble ordinary cows with short horns and yellow hair, but also have many characteristics of their own. Due to the nutritious quality of their meat, their strength, and their docility, Weining play a significant role in local farming and prosperity. In addition, the incidence of intestinal diseases is fairly low compared to other types of cattle, one of the most important reasons for local people keeping Weining cattle.

The normal intestine harbors over 100 trillion microorganisms including bacteria (98%), fungi (0.1%), viruses, protists, archaea, and these microbial communities (Liu J. et al., 2019; Li et al., 2021). The gut microbiota colonizing the intestinal tract forms a symbiotic relationship with the host and plays a vital role in maintaining nutrient intake, immune regulation, and intestinal barrier integrity. In addition, the gut microbiome is believed to be a biochemical transformation that exerts beneficial substances such as antimicrobial peptides, vitamins, and enzymes. Increasing evidence suggests that the diversity and composition of the gut microbiome have been linked to species and health, with species being the primary cause of the gut microbiota, followed by health status. However, to date, knowledge of the gut microbiota characteristics of Weining and Angus cattle is limited.

As is well known, diarrhea occurs in all animals, especially in newborns, and causes death in about half of the ruminants (Li et al., 2018; Bu et al., 2020). Calf mortality due to diarrhea remains very high in most countries, e.g., 17% in Germany and 5% in the United States (Urie et al., 2018; Eibl et al., 2021). Several studies have also indicated that intestinal microbial dysbiosis drives the development of diarrhea (Han et al., 2017; Shao et al., 2020). Healthy and balanced intestinal flora reduce the risk of diarrhea (Huang et al., 2019; Zuo et al., 2021). Previous studies have shown that intestinal bacteria in some ruminants alternate between dominant and weak populations with diarrheal symptoms (Yang et al., 2017). Thus, there may be some unavoidable links between the alteration of intestinal microbial communities and diarrhea. In the last few years, we have found that other types of cattle like Angus were more susceptible to diarrhea in Weining China. The mortality rate of other types of cattle was 9%, whereas in Weining cattle it was 3%. However, so far, there is little information about the relationship between diarrhea and gut microbiota in Angus cattle.

Regarding this phenomenon, we speculated about whether the low incidence of diarrhea in Weining cattle was related to intestinal flora. Possible reasons are that the Weining cattle’s ancestors lived in relative isolation after entering the mountains and developed individual gut flora. However, the characteristics of gut microbiota in Weining cattle remain unclear. Here we seek to investigate and compare the composition and variability of gut bacteria in Weining cattle (WN), healthy Angus cattle (An), and diarrheal Angus cattle (DA). In addition, although there have been recent studies on gut microbiota and diarrhea, very few studies have been conducted on Angus cattle, and our study aims to better understand how gut microbes affect organismal health, exploring potential pathogenic microbes. Meanwhile, we aimed to explore the potential bacteria associated with disease resistance. These findings will aid in the future development of dietary interventions that may resolve or prevent enteric and diarrheal diseases in ruminants.

## Materials and methods

### Sample acquisition

Samples were taken between June and August 2022 in Weining China, the peak period for diarrhea incidence in cattle. In total, 18 individual fresh fecal samples were taken from 6 Weining cattle, 6 healthy Angus cattle, and 6 diarrheal cattle. All the cattle were a half-year-old and had lived in the same conditions. Prior to sampling, all specimens are tested by a professional veterinarian to assess their health, and the samples collected are immediately placed in liquid nitrogen fixation and transported back to the laboratory in dry ice as soon as possible.

### 16S rRNA gene amplicon sequencing

DNA was extracted from 200 mg of feces using the QIAamp DNA Mini Kit (QIAGEN, Hilden, Germany). A fragment from the V3-V4 hypervariable region of the bacterial 16S rRNA gene was amplified using the linker primer 338F (ACTCCTACGGGAGGCAGCA) and the reverse primer 806R (GGACTACHVGGGTWTCTAAT). PCR reactions contained: 1 μL forward index primer (10 mM), 1 μL reverse index primer (10 mm), 1 μL 10 ng/μL DNA template, and 17 μL mixPfx AccuPrime master (Invitrogen, United States). The reaction conditions are as follows: initial denaturation at 95°C for 5 min, followed by 30 cycles of denaturation at 95°C for 30 s, annealing at 55°C for 30 s, and extension at 72°C for 1 min, and final elongation for 5 min at 72°C. The PCR amplification was performed in duplicate under the same conditions to ensure the accuracy of the results. In addition, we constructed the quality libraries with a single peak and concentration of more than 2 nM using a bioanalyzer (Agilent Technologies, United States) and quantitative PCR (qPCR). At last, the qualified library was sequenced on the Hiseq6000 platform (Illumina, United States), targeting the sequences with paired-end reads (Wang et al., 2022).

### Sequencing analysis

Quality screening of raw data generated by high-throughput sequencing using QIIME software (Qiime1.9.1). Questionable sequences such as short sequences (<200 bp), mismatched primers, and chimeras were removed. The resulting eligible sequences were segmented and clustered by OTU based on 97% similarity. The αlpha diversity indices of gut diversity were calculated based on the relative abundance distribution of OTUs in each sample. Meanwhile, βeta diversity indices were used to dissect the differences and similarities of the major components of the gut flora. Additionally, sparsity curves were generated for each sample to assess the sequencing depth. The data were statistically analyzed using GraphPad Prism (version 9.0c). Data are expressed as Mean ± SD, and *p* < 0.05 was considered statistically significant.

## Results

### Data acquisition and analysis

**Figure 1:**
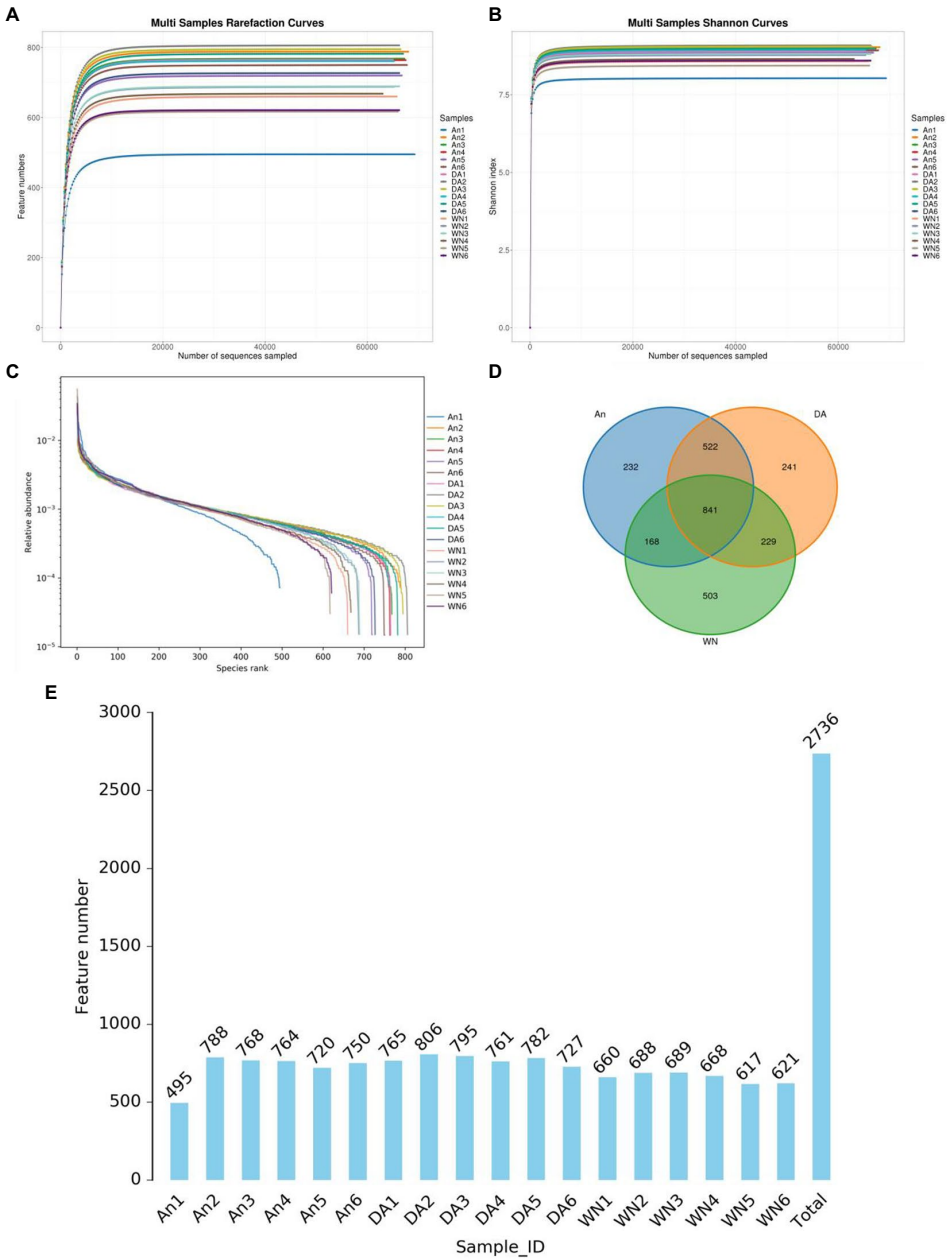
Feasibility analysis and OTUs distribution of amplicon sequencing. **(A,B)** Rarefaction curves. **(C)** rank abundance curves. **(D)** Venn diagram. **(E)** The numbers OTUs in each sample.

In the 16S rDNA high-throughput sequencing, 18 stool samples yielded a total of 1,441,028 raw sequences, of which the AN (healthy Angus), DA (Diarrheal Angus), and WN (Weining cattle) groups contained 480,043, 480,101, and 480,884 sequences, respectively (Table 1). The quality of the raw data was assessed and a total of 1,434,904 qualified sequences were obtained (An: 479260, DA: 479311, WN: 480104). Both the sparsity curve and the rank abundance curve showed a saturation trend, indicating that the depth and uniformity of sequencing could meet the requirements of the subsequent analysis (Figures 1A–C). The qualified sequences were clustered into 2,736 OTUs based on 97% nucleic acid sequence similarity, with the number of OTUs per sample ranging from 495 to 806 (Figures 1D,E). There were 841 shared OTUs in AN, DA, and WN groups and unique OTUs in each group were 232, 241, and 503, respectively.

**Table 1:** The bacterial sequence information of each sample.

Sample	Raw reads	Clean reads	Denoised reads	Merged reads	Effective reads	Effective (%)
An1	79,844	79,710	78,547	77,107	75,534	94.60%
An2	80,227	80,092	78,702	76,813	74,138	92.41%
An3	80,084	79,951	78,474	76,635	73,689	92.01%
An4	79,887	79,762	78,207	76,281	73,983	92.60%
An5	79,992	79,859	78,254	76,310	73,514	91.90%
An6	80,009	79,886	78,258	76,096	73,004	91.24%
DA1	80,230	80,100	78,572	76,355	73,208	91.25%
DA2	80,006	79,883	78,242	76,220	73,661	92.06%
DA3	80,116	79,980	78,366	76,194	73,243	91.42%
DA4	79,964	79,838	78,183	76,047	73,275	91.63%
DA5	79,968	79,837	78,214	75,943	73,470	91.87%
DA6	79,817	79,673	78,018	75,990	73,220	91.73%
WN1	80,221	80,084	78,432	76,051	73,622	91.77%
WN2	80,392	80,275	78,467	76,369	73,978	92.02%
WN3	80,091	79,977	78,176	75,869	73,714	92.03%
WN4	80,093	79,944	78,037	75,843	73,387	91.62%
WN5	79,942	79,823	78,093	75,738	72,924	91.22%
WN6	80,145	80,001	78,411	76,347	74,313	92.72%

### Comparative analysis of gut microbial diversity

**Figure 2:**
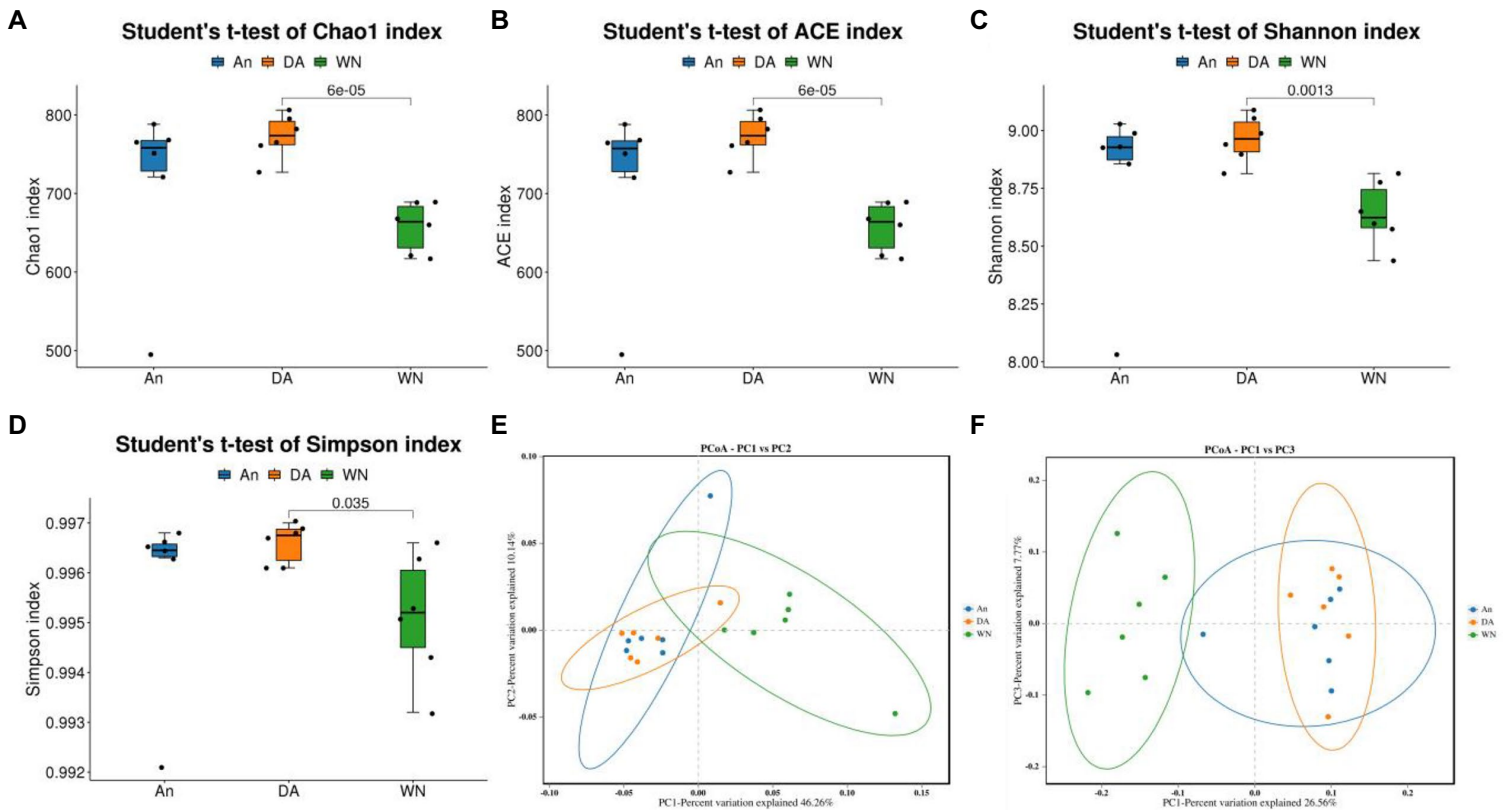
Changes of gut microbial diversity associated with species and diarrhea. **(A–D)** Chao1, ACE, Shannon, and Simpson indices. **(E,F)** PCoA plots based on the weighted and unweighted UniFrac distance.

Alpha analysis was applied to discover the difference in intestinal microbial community richness and diversity. The averages of the Shannon index were 8.64, 8.79, and 8.96 in the WN, An, and DA groups (*p* < 0.05). Moreover, there were no significant differences in the Chao1 (An = 714.67, DA = 772.67, WN = 657.25), Simpson (An = 0.99, DA = 0.99, WN = 0.99), and ACE (An = 714.40, DA = 772.78, WN = 657.29) index, indicating that the diversity of intestinal flora in groups WN, An and DA were not significantly different (Figures 2A–D). PCoA was applied to dissect the gut microbial variability and similarity among intergroup and intragroup individuals. The results of PCoA and NMDS showed that the samples from An and DA were clustered together and separated from the WN group, indicating that the intestinal flora composition of Weining cattle was different from Angus cattle, and the intestinal microbiota community diversity index was slightly affected by diarrhea in the An and DA groups (Figures 2E,F).

### Analysis of gut microbial community

**Figure 3:**
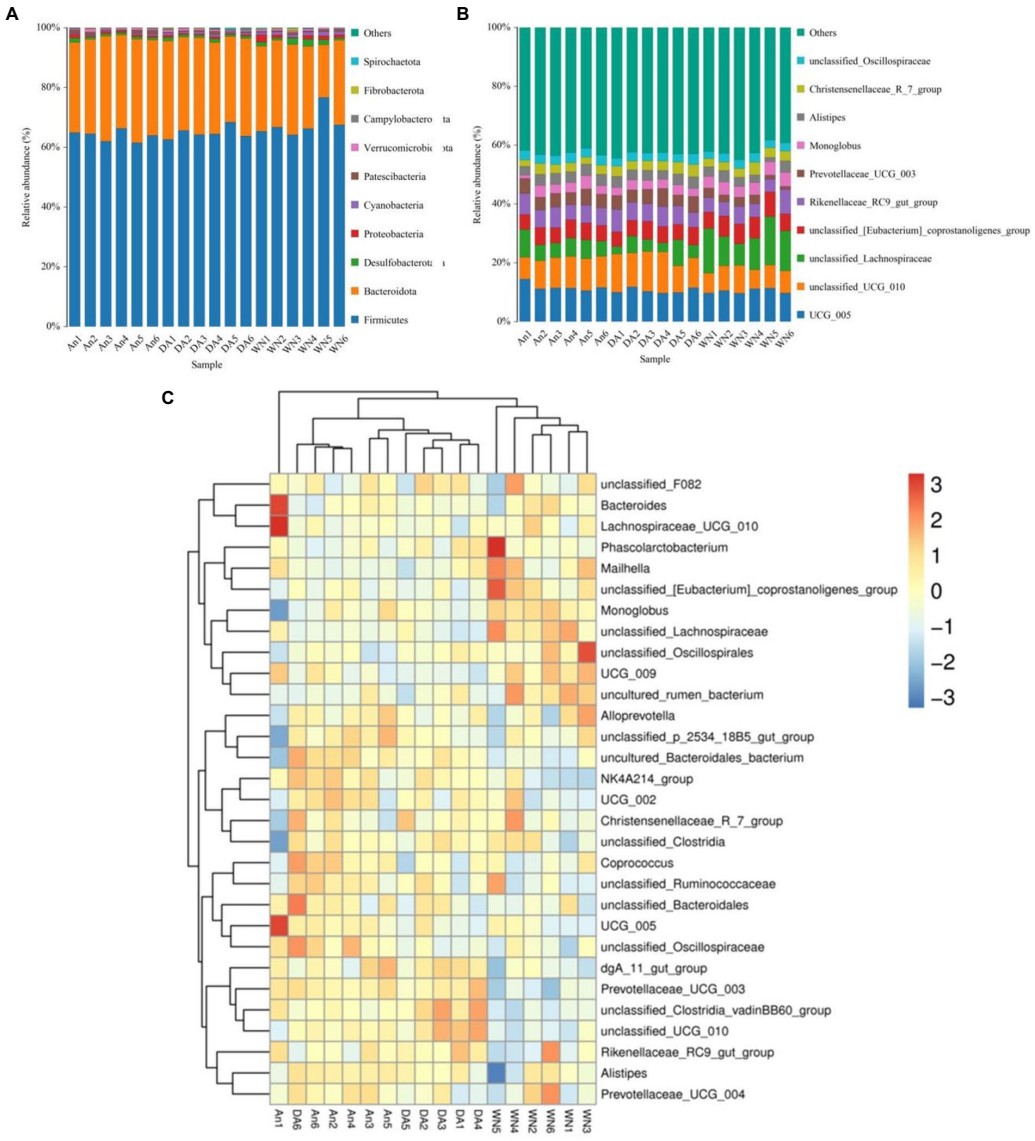
Changes of gut microbial composition associated with species and diarrhea. **(A,B)** Composition and the relative ratio of preponderant bacteria at the phylum and genus levels. **(C)** Heatmap of the 50 most abundant bacterial genera.

The relative proportions of different levels of sample-dominated flora were detected by microbial taxon assignment. At the phyla level, a total of 12 phyla were detected from all samples, ranging from 8 to 11 per sample. *Firmicutes* (63.89, 64.87, and 67.80%), *Bacteroidota* (32.42, 31.42, and 26.86%), *Desulfobacterota* (0.93, 0.93, and 1.52%) were the most abundant in the An, DA, and WN groups under phyla level, occupying more than 90% of all bacteria composition (Figure 3A). *Proteobacteria* (0.63, 0.50, and 1.22%), *Cyanobacteria* (0.38, 0.61, and 1.01%), *Fibrobacterota* (0.04, 0.13, and 0.23%), *Spirochaetota* (0.02, 0.12, and 0.09%), *Patescibacteria* (0.90, 0.49, and 0.57%), *Verrucomicrobiota* (0.55, 0.54, and 0.56%), and *Campylobacterota* (0.19, 0.32, and 0.01%) were observed with a lower abundance in the An, DA, and WN groups.

Among the genus identified, the *Lachnospiraceae* (12.23%) was notably enriched in the WT group, closely followed by UCG_005 (10.38), unclassified_UCG_010 (0.73%), *Rikenellaceae_RC9_gut_group* (0.55%), *unclassified_[Eubacterium]_coprostanoligenes_group* (6.8%), *Alistipes* (3.0%) and *Monoglobus* (4.3%). The results showed a very high proportion of beneficial bacteria in the Weining cattle.

In addition, the *Rikenellaceae_*RC9 (6.2 and 6.3%), UCG_005 (11.79 and 10.55%), *Lachnospiraceae* (6.29 and 4.82%), *unclassified_[Eubacterium]_coprostanoligenes_group* (5.57 and 5.56%), *Prevotellaceae_UCG_003* (4.74 and 4.86%), *Monoglobus* (3.12 and 3.09%), and *unclassified_Oscillospiraceae* (3.23 and 2.98%) were the main bacterial genus in An and DA groups (Figure 3B). The data indicated that most of the bacteria that are beneficial for health are significantly higher in the An group than in the DA group. The heatmap showed higher intra-group similarity and greater inter-group variability, revealing differences in gut microbiota composition between the An, DA, and WN groups (Figure 3C).

**Figure 4:**
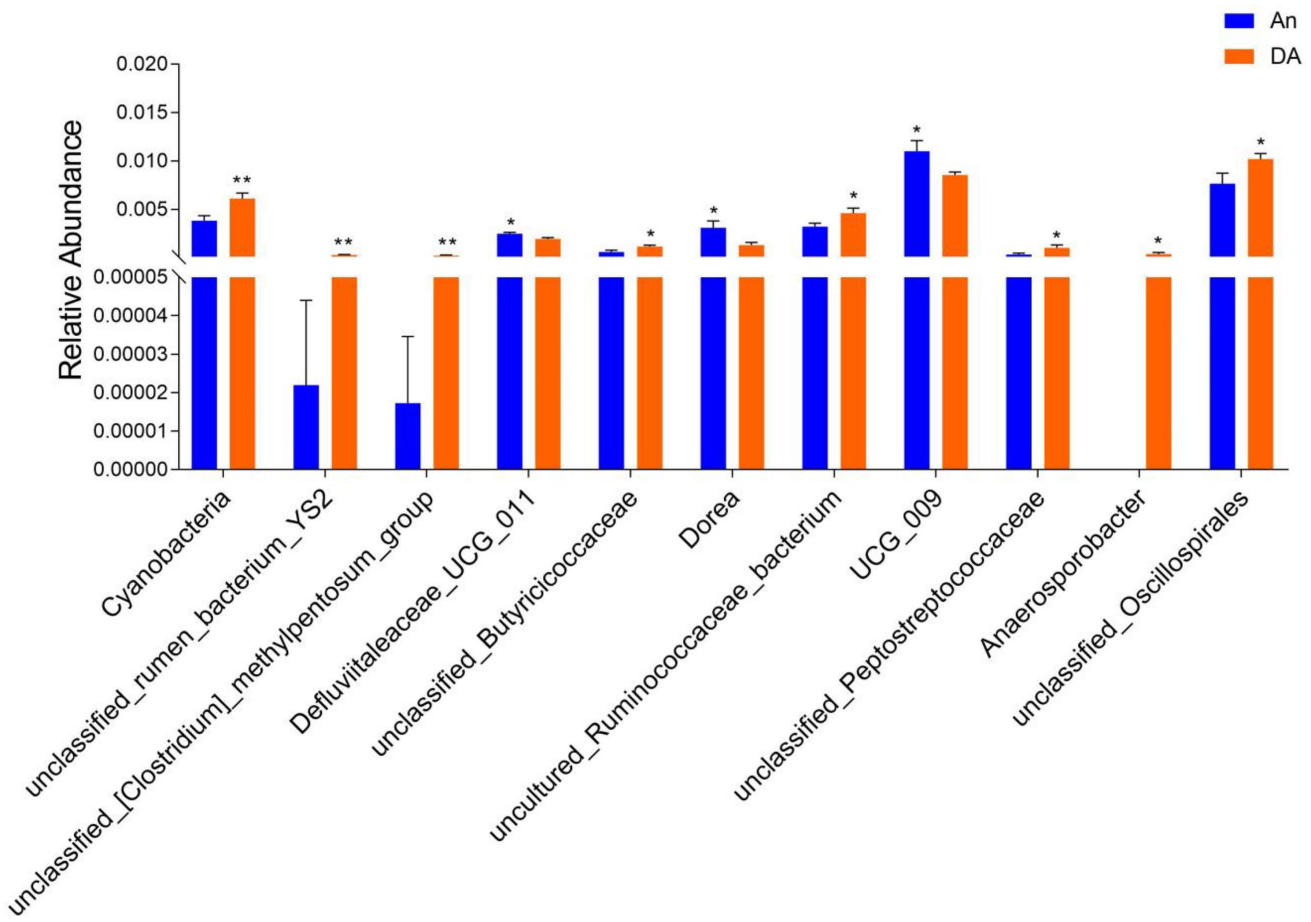
Statistical analysis of differential bacteria between An and DA groups at the phylum and genus levels.

**Figure 5:**
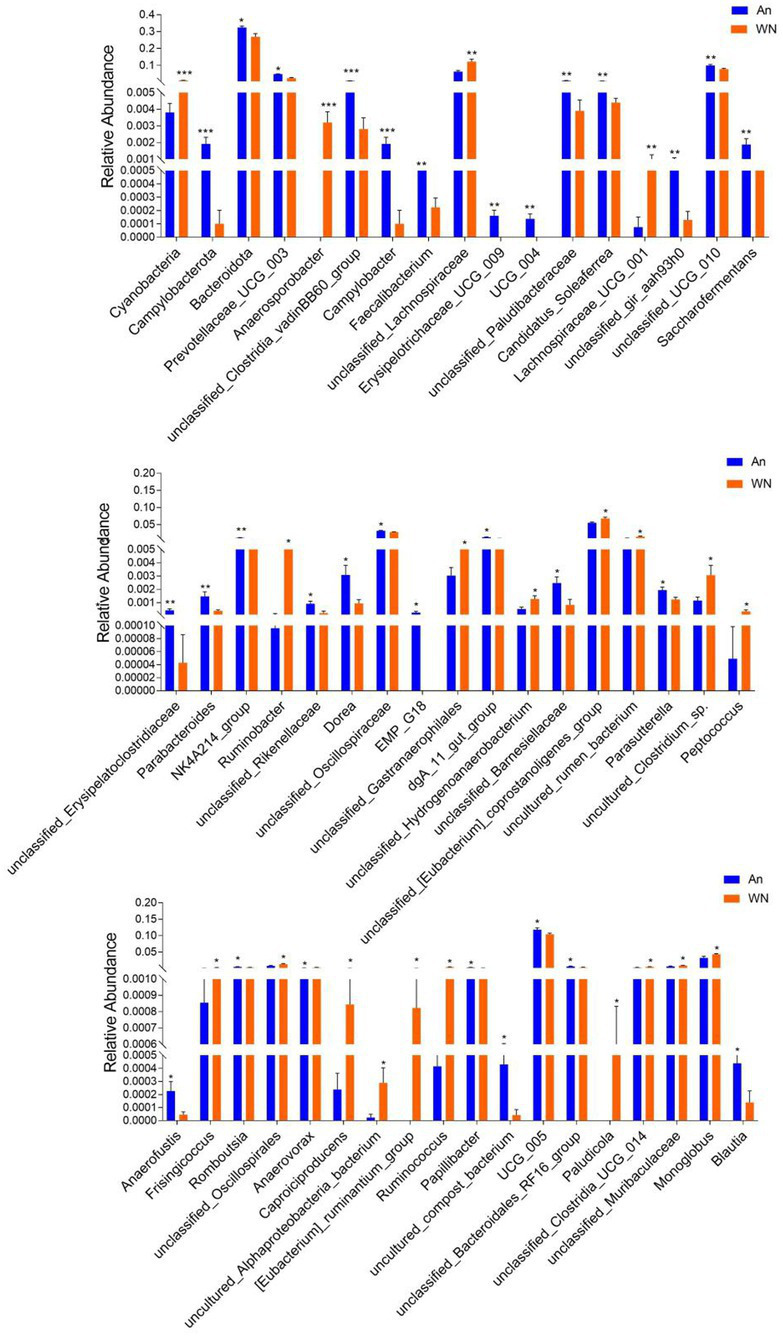
Statistical analysis of differential bacteria between An and WN groups at the phylum and genus levels.

**Figure 6:**
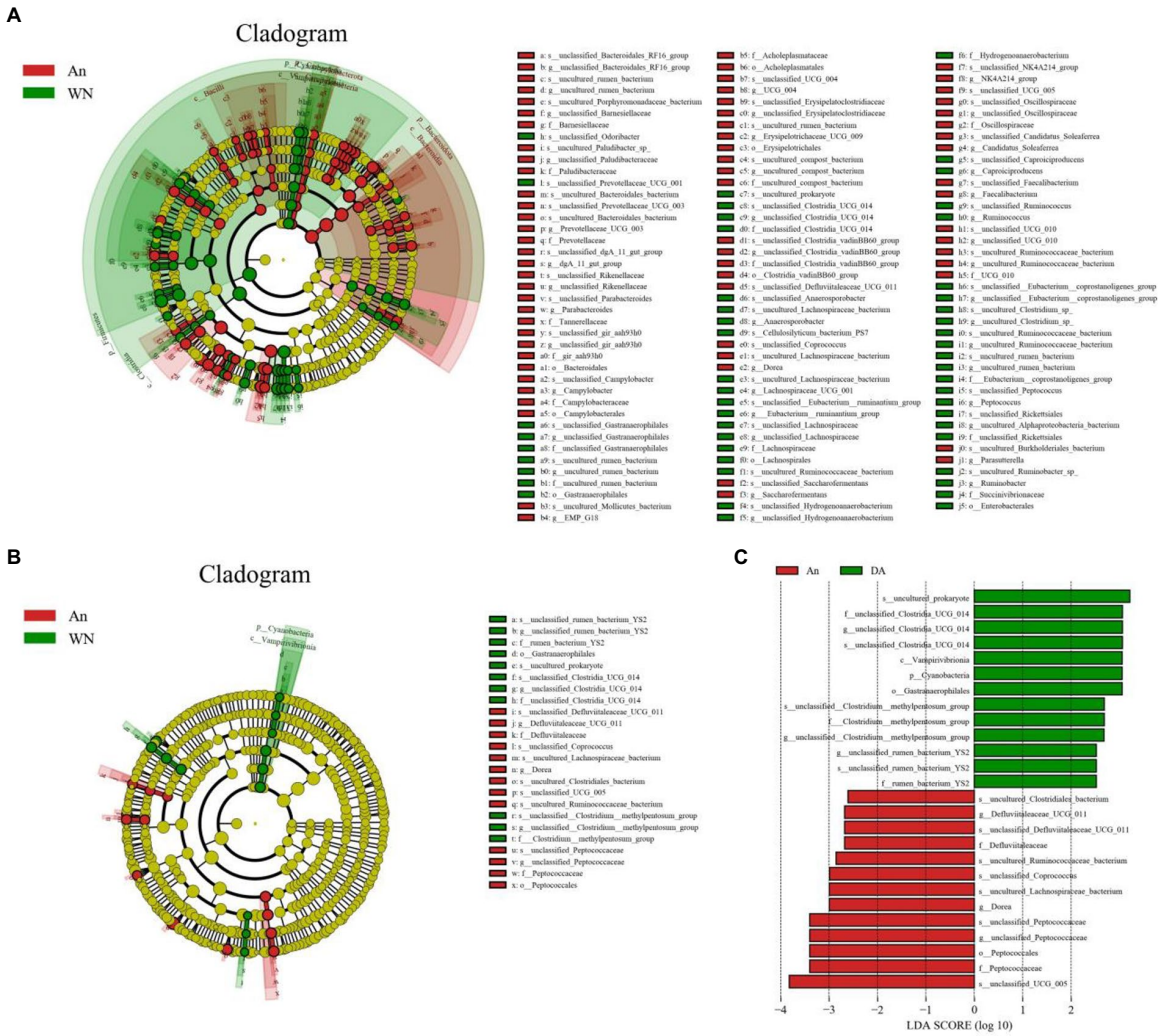
LEfSe integrated with LDA scores recognized differentially abundant taxon related to species and diarrhea. **(A,B)** Cladogram shows the phylogenetic distribution of differential taxon. **(C)** LDA scores >2 are considered significant.

Metastatic analysis was performed to explore differences in gut microbiota between the WN, An, and DA groups. A comparison of the An and DA groups showed a significant decline in the abundances of 7 genus (*unclassified_rumen_bacterium_YS2*, *unclassified_[Clostridium]_methylpentosum_group*, *unclassified_Butyricicoccaceae*, *uncultured_Ruminococcaceae_bacterium*, *unclassified_Peptostreptococcaceae*, *Anaerosporobacter*, and *unclassified_Oscillospirales*) as well as a significant increase in the abundances of 3 genus (*Defluviitaleaceae_UCG_011*, *Dorea*, and *UCG_009*; Figure 4). At the phyla level, the An group showed dramatically higher abundances of *Campylobacterota* and *Bacteroidota*, whereas the WN group enriched for *Cyanobacteria* and *Elusimicrobiota*. Compared with the An group, the gut microbiota in the WN group showed a distinct decrease in the relative abundances of *Prevotellaceae_UCG_003*, *unclassified_Clostridia_vadinBB60_group*, *Campylobacter*, *Faecalibacterium*, *Erysipelotrichaceae_UCG_009*, *UCG_004*, *unclassified*_*Paludibacteraceae*, *Candidatus_Soleaferrea*, *unclassified_gir_aah93h0*, *unclassified_UCG_010*, *Saccharofermentans*, *unclassified*_*Erysipelatoclostridiaceae*, *Parabacteroides*, *NK4A214*_*group*, *unclassified_Rikenellaceae*, *Dorea*, *unclassified_Oscillospiraceae*, *EMP_G18*, *dgA_11_gut_group*, *unclassified_Barnesiellaceae*, *Parasutterella*, *Anaerofustis*, *Romboutsia*, *Papillibacter*, *uncultured*_*compost*_*bacterium*, *UCG_005*, *unclassified_Bacteroidales_RF16_group* and *Blautia*, whereas *Anaerosporobacter*, *unclassified_Lachnospiraceae*, *Lachnospiraceae_UCG_001*, *Ruminobacter*, *unclassified_Gastranaerophilales*, *unclassified_Hydrogenoanaerobacterium*, *unclassified_[Eubacterium]_coprostanoligenes_group*, *uncultured_rumen_bacterium*, *uncultured_Clostridium_sp., Peptococcus*, *Frisingicoccus*, *unclassified_Oscillospirales*, *Anaerovorax*, *Caproiciproducens*, *uncultured_Alphaproteobacteria_bacterium*, *[Eubacterium]_ruminantium_group*, *Ruminococcus*, *Paludicola*, *unclassified_Clostridia_UCG_014*, *unclassified_Muribaculaceae*, and *Monoglobus* increased significantly (Figure 5). Moreover, the cladogram was generated by applying LefSe to further investigate variability in bacterial taxa composition. In addition to the significantly different bacteria mentioned above, we observed that several bacteria such as *Campylobacteria* and *Anaerosporobacter* were reached in the DA group, whereas beneficial bacteria such as *Lachnospiraceae* were significantly overrepresented in the WN group (Figure 6).

### Correlation network analysis

**Figure 7:**
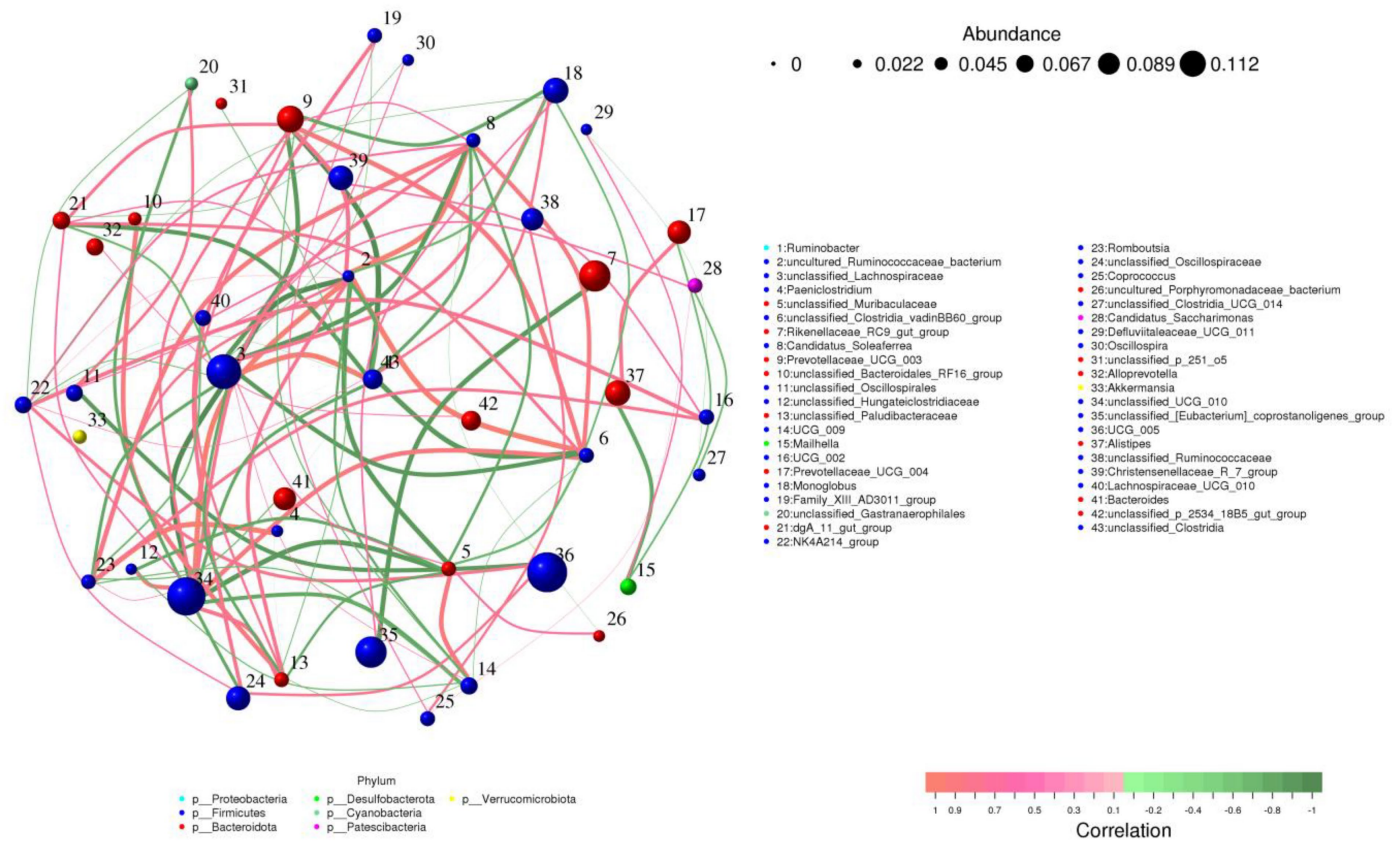
Correlation network analysis of gut microbiota. Positive and negative correlations are indicated by red and green lines, respectively.

*Prevotellaceae_UCG_003* was positively associated with *unclassified_UCG_010*. *Ruminobacter* was negatively related to *dgA_11_gut_group*, *uncultured_Ruminococcaceae_bacterium*, *unclassified_UCG_010*, *UCG_009*, *NK4A214_group*, *unclassified_Clostridia_vadinBB60_group*, *Prevotellaceae_UCG_003* but positively associated with *unclassified_Lachnospiraceae, UCG_002* and *Monoglobus* (Figure 7).

## Discussion

Diarrhea is the most common disease in calves and severely affects the animal’s growth and development. According to previous reports, the economic damage caused by diarrhea is enormous and difficult to control (Fischer et al., 2016; Lorenz et al., 2021). Studies indicated that the etiology of bovine diarrhea is multifactorial, with pathogens and management factors (housing, feeding, and sanitary conditions) playing an important role (Bendali et al., 1999). A study in diarrheal calves in Belgium estimated the prevalence of *E. coliRotavirus*, *Coronavirus*, and *C. parvum* at 4, 20, 8, and 31%, respectively. In a recent Swiss study on diarrheal calves, the prevalence of these *Enteropathogens* remained at high levels. Moreover, diarrhea inevitably accompanies intestinal damage, suggesting that the intestinal microbiota may be altered (Xia et al., 2018; Zhai et al., 2019). As the habitat of intestinal flora, the gastrointestinal tract is more susceptible to various diseases such as inflammatory bowel disease and diarrhea due to the influence of intestinal flora (Liu C. S. et al., 2019; Yue et al., 2019; Zhang et al., 2022). In addition, although the gut microbiota inhabits the gut, significant changes in the gut microbiota may also lead to the development of other diseases such as liver disease, diabetes, and obesity, etc. (Guo et al., 2022; Ye et al., 2022). More importantly, significant changes in gut microbiota also affect gut permeability, which may lead to a leaky gut and increased rates of pathogenic bacterial infection (Yue et al., 2020; Xu et al., 2021). Therefore, the characterization of gut microbiota is crucial for the prevention, control, and diagnosis of diarrhea (Bjorkman et al., 2003; Singh et al., 2015).

Gut microbial diversity and richness constantly decrease under the influence of diarrhea (Zhang L. et al., 2020; Cui et al., 2021; Ren et al., 2022). In our study, we found no significant difference in gut microbiota diversity and richness between the An group and DA group, the lack of difference is most likely due to an increase in pathogenic bacteria due to diarrhea and a decrease in beneficial bacteria in the DA group. In Han’s report, there was no significant difference in gut microbial diversity between healthy and diarrheal yaks, which was consistent with our findings (Han et al., 2017). Similarly, He′s results are also similar to ours: diarrhea does not significantly alter the diversity and richness of gut microbiota in pigs (He et al., 2020). Although the diversity and richness of intestinal microbiota did not change significantly, the composition of bacteria did. *Anaerosporbacter* were rich in the DA group but were not detected in the An group. *Anaerosporbacter* is likely to be associated with the occurrence of colorectal cancer. In Yu’s study, the results manifested that the *Anaerosporbacter* were abundant in the colorectal cancer group compared to the healthy group (Yu et al., 2017). *Campylobacter* is recognized as the most common cause of bacterial enteritis (Liu et al., 2018). Among bacterial infections reported in recent years, *Campylobacter* spp. predominated. In Singh’s report, *Campylobacter* usually causes asymptomatic infections, diarrhea, and hemorrhagic colitis (Singh et al., 2015; Shin et al., 2021). Interestingly, the aforementioned potentially pathogenic bacteria exhibited a significant relative abundance in the DA group compared to the An group. *Candidatus Soleaferrea* secretes homeostatic protective properties and has anti-inflammatory effects (Zhang et al., 2015), there was no significant difference in the proportion of *Candidatus Soleaferrea* in the An and DA groups. Conversely, the beneficial bacteria including *Dorea*, *Muribaculaceae, UCG-009*, and *Monoglobus* were significantly lower in the DA group compared with healthy Angus cattle. *Monoglobus* is a beneficial bacteria that modulates the metabolism (Kim et al., 2019). Unique among known human gut flora, *Monoglobus* plays an active role in pectin degradation and sugar utilization (Kim et al., 2019). *Muribaculaceae* can produce propionate, which is closely related to gut health. Related studies have reported that *Muribaculaceae* are closely related to acarbose consumption (Smith et al., 2021). In addition, *Dorea* and *UCG-009* are capable of regulating health and absorbing nutrients. In the current study, the gut microbiota of Angus cattle with diarrhea was significantly altered, implying an imbalance in gut homeostasis. Our study showed that gut microbiota dysbiosis is an important factor driving the development of diarrhea. At the same time, the findings shed light on potential pathogens including *Anaerosporbacter* and *Campylobacter*, which cause diarrhea in Angus cattle.

It is well known that gut microbiota are an important indicator for evaluating gut function and homeostasis (Xia et al., 2018; Duan et al., 2020; Reese et al., 2021). However, the diversity of gut microbiota is easily affected by various factors such as species, age, and various diseases (Ding et al., 2019; Wu et al., 2020, 2022). Species are the most important factor affecting gut microbiota (Yang et al., 2018; Huang et al., 2020; Zhong et al., 2021). Animals of various species need to evolve different intestinal flora structures to adapt to their habitats, and diet characteristics, etc. (Liu et al., 2021, 2022). For example, herbivores have a higher intestinal flora structure to digest cellulose and realize energy conversion (Dong et al., 2020; Yang et al., 2022). In addition, different breeds of sheep and chickens also have different intestinal flora structures (Rettedal et al., 2019; Qin et al., 2020). Captive horses, for example, have lower gut microbiota composition and lower numbers of pathogenic bacteria than wild donkeys (Zhou et al., 2022). In addition, compared with cattle living in plain areas, yaks need to evolve a more diverse intestinal flora structure to adapt to the high-altitude hypoxic environment of the Qinghai-Tibet Plateau (Fu et al., 2021; Wang et al., 2021). Intestinal flora are executors of intestinal function and the supervisor of intestinal health, so changes in intestinal flora also affect the health of the host (Yin et al., 2019; Zhang P. et al., 2020; Chen et al., 2022).

Weining cattle are a native breed in Guizhou, while Angus cattle are an exotic breed introduced to Weining in the last 10 years. Weining cattle and Angus cattle are currently the main breeds in Weining Guizhou, occupying more than 90% of the local cattle industry. In the past 3 years, the incidence of diarrhea was less than 3% in Weining cattle. However, the rate of diarrhea in other breeds of cattle was significantly higher than 5%. Previous studies have demonstrated that the gut microbiome is correlated with species and health. In some studies, greater quantities of *Firmicutes* were found in the WN group compared to the An groups. Previous research has reported that *Firmicutes* are closely related to the health of gut microbiota (Eckburg et al., 2005), which contribute to maintaining gut microbiota balance, regulating the gut environment, and inhibiting pathogens (Sun et al., 2016). In addition, *Lachnospiraceae*, *Rikenellaceae*, and *Coprostanoligenes* were the most dominant genus in the WN group. *Lachnospiraceae* is closely linked to host health by producing short-chain fatty acids, converting primary to secondary bile acids, and inhibiting intestinal pathogens (Sorbara et al., 2020). The *Rikenellaceae* and *Coprostanoligenes* were regarded as a beneficial bacterium in modulating health and serum dyslipidemia (Tavella et al., 2021). *Rikenellaceae* plays an essential role in maintaining intestinal mucosal immunity, Previous studies have demonstrated that HIV infection is distinctly involved with the loss of *Rikenellaceae* (Dubourg et al., 2017). Similarly, in Teresa Tavella’s research, *Rikenellaceae* could significantly reduce visceral adipose tissue and help maintain a healthier metabolic profile, which proved that adequate *Rikenellaceae* could improve the body’s health and metabolism (Backhed et al., 2015; Dubin et al., 2016). Meanwhile, Wei et al. (2021) reported that *Coprostanoligenes* have the ability to modulate serum dyslipidemia. *Overall*, the highest abundance of beneficial bacteria was present in the WN group compared to the An and DA groups. In particular, the *Lachnospiraceae* are over-represented in Weining cattle, showing significantly higher abundances compared to Angus cattle. Interestingly, the *Muribaculaceae*, which plays an important role in anti-inflammatory action was also enriched in the WN group. Conversely, the potential pathogens including *Alistipes* and *Campylobacteria* were lower present in the WN group compared to the An group. Overall, the greater abundance and diversity of beneficial bacteria indicated the potential of Weining cattle for diarrhea prevention and health modulation. The data revealed that a good gut microbiome structure improves the body’s disease resistance and health status. In addition, Weining cattle have great potential as an isolated source of probiotics.

## Conclusion

This study characterized the gut microbiota diversity and composition in Weining cattle and Angus cattle. The WN group had a greater abundance of beneficial bacteria and a lower abundance of potential pathogens. While there was no significant difference between healthy Angus and diarrheal Angus, there was a significant change in the type and proportion of bacteria. The potential pathogens including *Anaerosporbacter* and *Campylobacter* were higher in diarrheal cattle, conversely, the beneficial bacteria including *Dorea*, *Muribaculaceae, UCG-009*, and *Monoglobus* were significantly lower compared to healthy cattle. This is the first report of gut microbiota in Weining cattle and broadens the knowledge of gut microbiota. Our results convey the message that diarrhea not only directly modifies the diversity and abundance of gut microbiota but also indirectly affects some functional bacteria. In addition, this study revealed potentially pathogenic bacteria and provided basic data for the subsequent treatment of diarrhea in Angus cattle.

## Data availability statement

The datasets presented in this study can be found in online repositories. The names of the repository/repositories and accession number(s) can be found at: https://www.ncbi.nlm.nih.gov/, PRJNA931445.

## Ethics statement

The study was conducted under the guidance and approval of the Animal Welfare and Ethics Committee of Huazhong Agricultural University.

## Author contributions

LW, JM, and MW provided the research idea. DW, JM, and MW contributed reagents, materials, and analysis tools. LW wrote the manuscript. KL and YZ revised the manuscript. All authors participated in writing and reviewing the manuscript, contributed to the article and approved the submitted version.

## Funding

The study was supported by Research and Demonstration of Key Technology for Nutritional Control and Efficient Utilization of Roughage for Weining Cattle [Guizhou Science (2021) General project No. 153], the Weining Cattle Breeding Base of Weining County, Guizhou Province, and the Sixth Batch of Talent Base Project of Guizhou Province [Guizhou people Hair Collar (2018) No. 3].

## Conflict of interest

The authors declare that the research was conducted in the absence of any commercial or financial relationships that could be construed as a potential conflict of interest.

## Publisher’s note

All claims expressed in this article are solely those of the authors and do not necessarily represent those of their affiliated organizations, or those of the publisher, the editors and the reviewers. Any product that may be evaluated in this article, or claim that may be made by its manufacturer, is not guaranteed or endorsed by the publisher.
