# Supplementary figures and images for: Dynamic distribution of gut microbiota in cattle at different breeds and health states
Source: Front Microbiol. 2023 Feb 16;14:1113730. doi: 10.3389/fmicb.2023.1113730 (PMC9978850; doi:10.3389/fmicb.2023.1113730)

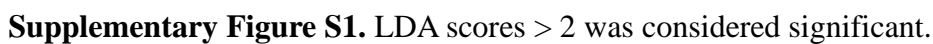

Supplement: Supplementary file 1 [file Image_1.pdf]
